# Tissue Plasminogen Activator and Plasminogen Activator Inhibitor 1 Contribute to Sonic Hedgehog-Induced In Vitro Cerebral Angiogenesis

**DOI:** 10.1371/journal.pone.0033444

**Published:** 2012-03-14

**Authors:** Hua Teng, Michael Chopp, Ann Hozeska-Solgot, Lihong Shen, Mei Lu, Clark Tang, Zheng Gang Zhang

**Affiliations:** 1 Department of Neurology, Henry Ford Hospital, Detroit, Michigan, United States of America; 2 Department of Physics, Oakland University, Rochester, Michigan, United States of America; 3 Department of Biostatistics and Research Epidemiology, Henry Ford Hospital, Detroit, Michigan, United States of America; University of South Florida, United States of America

## Abstract

The molecular mechanisms underlying cerebral angiogenesis have not been fully investigated. Using primary mouse brain endothelial cells (MBECs) and a capillary-like tube formation assay, we investigated whether the sonic hedgehog (Shh) signaling pathway is coupled with the plasminogen/plasmin system in mediating cerebral angiogenesis. We found that incubation of MBECs with recombinant human Shh (rhShh) substantially increased the tube formation in naïve MBECs. This was associated with increases in tissue plasminogen activator (tPA) activation and reduction of plasminogen activator inhibitor 1 (PAI-1). Blockage of the Shh pathway with cyclopamine abolished the induction of tube formation and the effect of rhShh on tPA and PAI-1. Addition of PAI-1 reduced rhShh-augmented tube formation. Genetic ablation of tPA in MBECs impaired tube formation and downregulated of vascular endothelial growth factor (VEGF) and angiopoietin 1 (Ang1). Addition of rhShh to tPA−/− MBECs only partially restored the tube formation and upregulated Ang1, but not VEGF, although rhShh increased VEGF and Ang1 expression on wild-type MBECs. Complete restoration of tube formation in tPA−/− MBECs was observed only when both exogenous Shh and tPA were added. The present study provides evidence that tPA and PAI-1 contribute to Shh-induced in vitro cerebral angiogenesis.

## Introduction

Stroke induces angiogenesis during the process of brain repair, and angiogenesis in the ischemic brain is related to improvement of functional outcome [1], [2], [3], [4]. Angiogenesis consists of a series of endothelial cellular events, including proliferation, migration, formation of tube like structures, and maturation into new blood vessels [5], [6]. Molecular mechanisms underlying cerebral angiogenesis have not been extensively investigated.

Sonic hedgehog (Shh), a member of the family of the hedgehog proteins [7], mediates angiogenesis during development [8], [9] and under pathological conditions [10], [11], [12]. Shh binds to its transmembrane receptor Patched (Ptch) [12] that results in the activation of the transmembrane protein smoothened (Smo), and subsequently triggers an intracellular signal transduction pathway that leads to the activation of the Gli transcription factors [7]. Shh has been implicated in the induction of cerebral angiogenesis [13], [14]. Shh mediates neural tube angiogenesis during embryonic development [14]. In vitro, Shh induces capillary tube formation in a murine brain capillary endothelial cell line [13].

Plasminogen activators, tPA and urokinase plasminogen activator (uPA), convert plasminogen into plasmin [15]. Plasmin regulates angiogenesis directly by degrading matrix molecules and indirectly by activating extracellular matrix metalloproteinases and angiogenic growth factors [16], [17]. Plasminogen activator inhibitor 1 (PAI-1) also plays a critical angiogenic role [18], [19], [20]. In the cerebral circulation, cerebral endothelial cells are primary contributors of tPA and PAI-1 [21].

We and others have recently demonstrated that astrocytes secrete Shh and cerebral endothelial cells express Ptch, Smo, and Gli1 [22], [23]. Thus, the possibility arises that activation of the Shh signaling pathway in cerebral endothelial cells by secreted Shh interacts with tPA and/or PAI to induce angiogenesis. Accordingly, we hypothesized that the interaction between the Shh signaling pathway and the plasminogen and plasmin system mediates cerebral angiogenesis. In the present study, using primary mouse brain endothelial cells (MBECs) and a capillary-like tube formation assay, we tested this hypothesis. Our data show that tPA and PAI-1 are required for Shh-induced cerebral angiogenesis, thereby suggesting that their crosstalk plays an important in cerebral angiogenesis.

## Results

### The Shh pathway promotes capillary-like tube formation in cerebral endothelial cell

**Figure 1:**
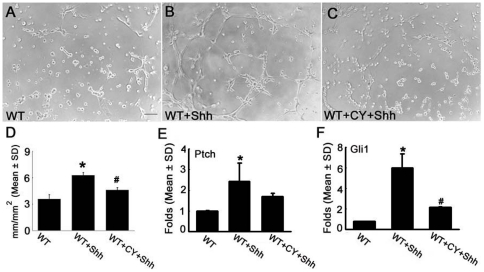
Shh increases capillary-like tube formation in primary cerebral endothelial cells isolated from wild-type mice. Panels A to C show representative images of capillary-like tube formation of primary cerebral endothelial cells incubated with control medium (A), rhShh (B), and rhShh in presence of cyclopamine (C). Panel D shows quantitative data of capillary-like tube lengths in mm/mm^2^ (n = 6/group). Real-time RT-PCR analysis (n = 3/group) showed that rhShh substantially increased mRNA levels of Ptch (E), a Shh receptor, and Gli 1(F), a transcription factor in endothelial cells. *p<0.05 the control group, # p<0.05 versus the rhShh group. Bar = 100 µm. WT = wild-type. CY = cyclopamine.

Using a capillary tube formation assay, we examined the effect of Shh on in vitro angiogenesis. Incubation of primary MBECs with rhShh (100 ng/ml) in Matrigel for 16 h significantly increased the number of tubes compared with the number in the control group (Fig. 1A, B, and D). We then examined whether rhShh affects its receptor and Gli1 expression on the MBECs. Quantitative real-time RT-PCR analysis revealed that treatment of MBECs with rhShh upregulated mRNA levels of Ptch, a Shh receptor, and Gli1, a transcription factor (Fig. 1E, F, and Figure S1 A to D). Blockage of the Shh pathway with a specific inhibitor of the Smo, cyclopamine (5 µM), abolished the rhShh-increased tube formation (Fig. 1C, D). These data indicate that exogenous Shh promotes angiogenesis in naive cerebral endothelial cells.

### tPA and PAI-1contribute to Shh-enhanced capillary tube formation

**Figure 2:**
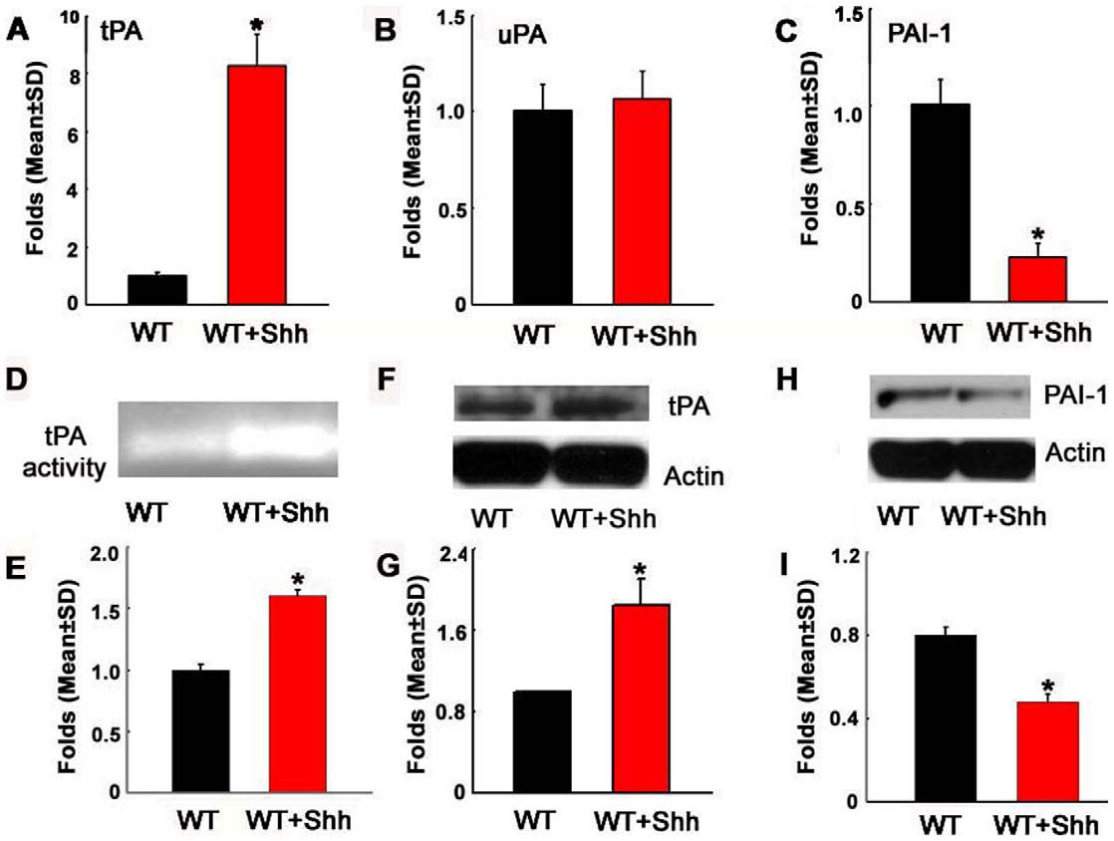
Shh increases tPA activity and expression by upregulating tPA expression and downregulating PAI-1 expression. Real-time RT-PCR analysis (n = 3/group) revealed that incubation of endothelia cells with rhShh significantly increased tPA mRNA levels (A) and reduced PAI-1 mRNA (C), but did not alter uPA mRNA levels (B). Zymography and Western blot analysis (n = 3/group) showed that rhShh significantly increased PA activity (D and E), protein expression (F and G), and reduced PAI-1 proteins (H and I), respectively. *p<0.05 compared to the control group. WT = wild-type.

To examine the effect of rhShh on the plasminogen/plasmin system, we measured mRNA and protein levels of genes in this system. Quantitative real-time RT-PCR analysis of naïve MBECs revealed that rhShh significantly upregulated tPA (Fig. 2A), but not uPA expression (Fig. 2B) and downregulated PAI-1 expression (Fig. 2C) compared with the endothelial cells in the control group. In parallel, zymography and Western blot analyses showed that rhShh significantly increased tPA activity (Fig. 2D, E) and protein levels (Fig. 2F, G), and substantially decreased PAI-1 protein levels (Fig. 2H, I). Blockage of Smo with cyclopamine suppressed rhShh-upregulated tPA expression (2.3±0.2 vs 8.3±1.1 in rhShh, n = 3/group, p<0.05) and restored PAI-1 expression (1.3±0.1 vs 0.2±0.07 in rhShh, n = 3/group, p<0.05). These data indicate that the Shh signaling pathway affects the plasminogen/plasmin system by activation of tPA and downregulation of PAI-1 expression on MBECs.

**Figure 3:**
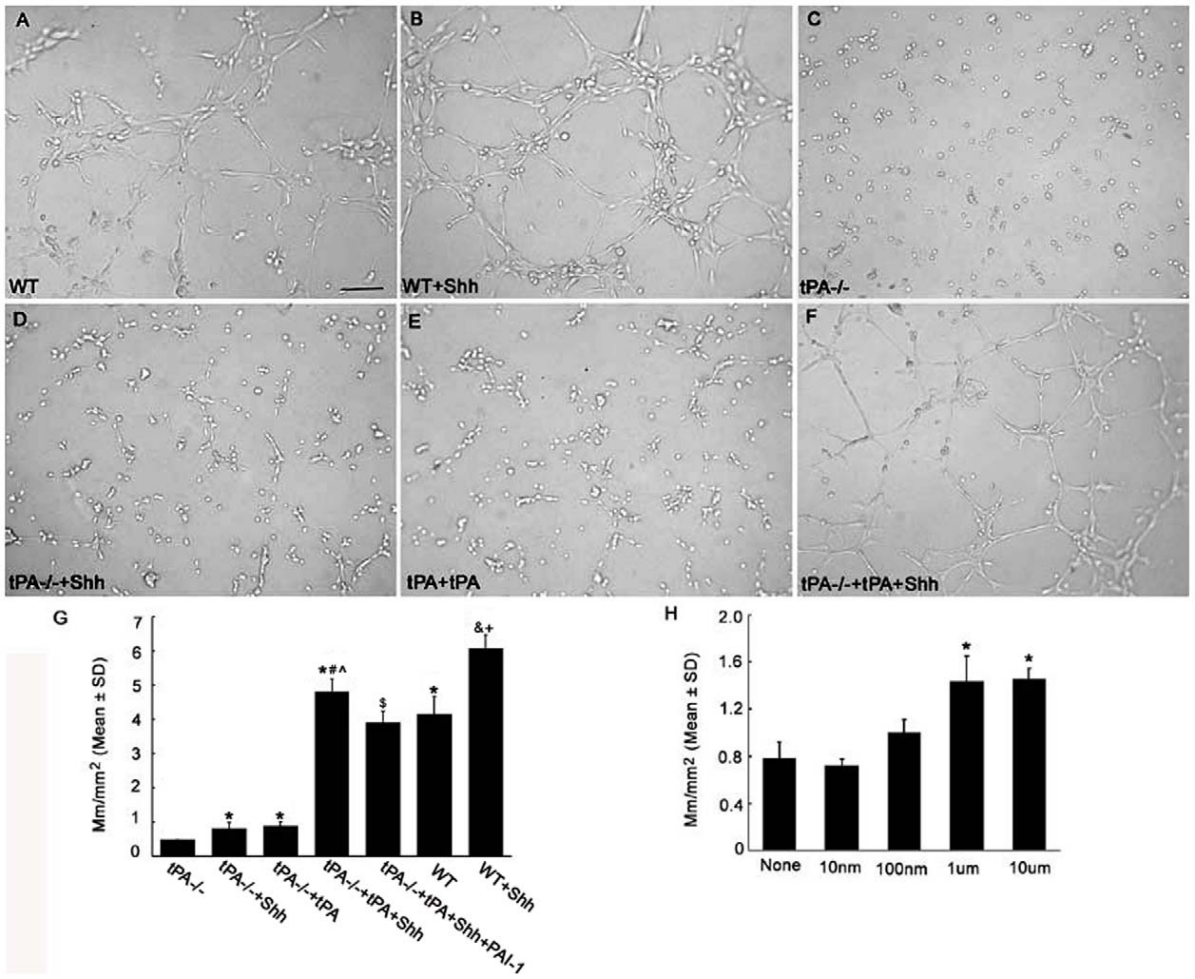
tPA activity partially mediated Shh-enhanced capillary tube formation. Panels A to F show representative images of capillary-like tube formation of primary cerebral endothelial cells incubated with control medium (A), rhShh (B), tPA−/−cerebral endothelial cells (C), tPA−/−cerebral endothelial cells incubated with rhShh (D), tPA−/−cerebral endothelial cells incubated with htPA (E), and), tPA−/−cerebral endothelial cells incubated with rhShh and htPA (F). Panel G shows quantitative data of capillary-like tube lengths in mm/mm^2^ among different experimental groups (n = 6/group). Panel H shows the effect of different doses of exogenous tPA on capillary-like tube formation on tPA−/− cerebral endothelial cell (n = 6/group). *p<0.05 versus the tPA−/−group, # p<0.05 versus the tPA−/−+tPA group, ∧p<0.05 versus the tPA−/−+Shh group, $ p<0.05 vs the tPA−/−+tPA+Shh group, & p<0.05 versus the WT group, and + p<0.05 versus the tPA−/−+tPA+Shh group. Bar = 100 µm. WT = wild-type. tPA−/− = tPA knockout.

We next examined whether activation of tPA by rhShh is required for rhShh-induced capillary tube formation by employing primary cerebral endothelial cells harvested from tPA−/− mice. Quantitative real-time RT-PCR analysis of the tPA−/− cerebral endothelial cells showed that tPA mRNA levels were almost undetectable (0.07±0.01, n = 3), while uPA levels were not altered (1.1±0.2, n = 3) compared to levels on wild-type endothelial cells (1.0±0.1, n = 3). The capillary tube formation assay showed that the tPA−/− endothelial cells exhibited an 87% reduction of capillary-like tube formation (Fig. 3C, G) compared to wild-type endothelial cells (Fig. 3A, G). To examine whether exogenous tPA can restore capillary tube formation of the tPA−/− endothelial cells, rhtPA was added. Surprisingly, exogenous rhtPA only modestly restored capillary tube formation of the tPA−/− endothelial cells in a dose-dependent manner (Fig. 3E, G, and H). Addition of rhShh into the tPA−/− endothelial cells also modestly increased the tube formation (Fig. 3D, G) compared with the tPA−/− endothelial cells (Fig. 3C, G). However, incubation of the tPA−/− endothelial cells with both rhtPA and rhShh fully restored the tube formation, which significantly surpassed tube formation levels of wild-type endothelial cells (Fig. 3F, G). These data suggest that tPA and Shh contribute to in vitro cerebral angiogenesis.

**Figure 4:**
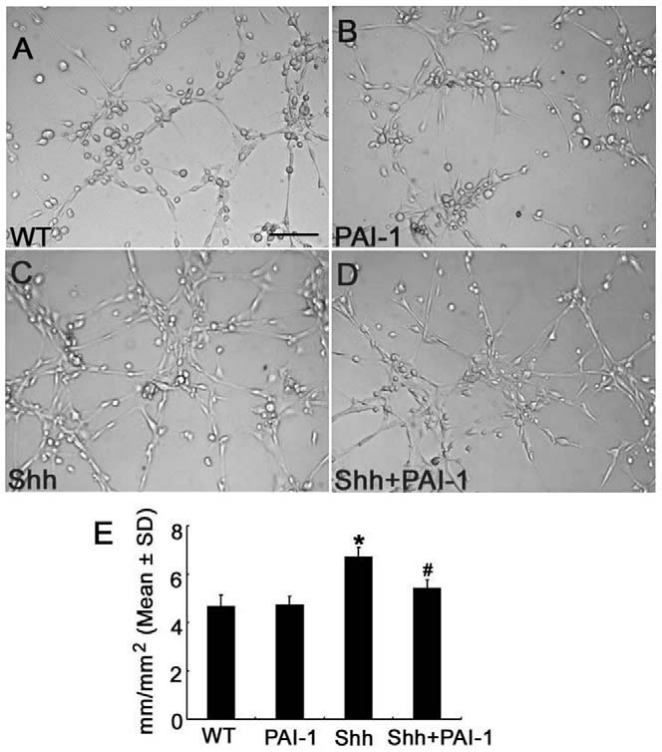
PAI-1 decreased Shh induced tube formation. Panels A to D show representative images of capillary-like tube formation of primary cerebral endothelial cells incubated with control medium (A), PAI-1(B), rhShh (C), and rhShh with PAI-1(D). Panel E shows quantitative data of capillary-like tube lengths in mm/mm^2^ among different experimental groups (n = 5/group).* p<0.05 the control group. # p<0.05 versus the rhShh group. Bar = 100 µm.

To examine whether PAI-1 contributes to Shh-induced tube formation, we incubated wild-type endothelial cells with PAI-1 (10 µg/ml) in the presence of rhShh and found that exogenous PAI-1 significantly reduced rhShh-increased tube formation (Fig. 4C, D and E), although exogenous PAI-1 alone did not affect baseline tube formation (Fig. 4A, B, and E). Furthermore, addition of PAI-1 into tPA−/− endothelial cells treated with rhtPA and rhShh partially, but significantly reduced tube formation (Fig. 3G). These suggest that PAI-1 is also involved in Shh-induced angiogenesis.

### Upregulation of Ang1 and VEGF by Shh is tPA dependent and independent, respectively

**Figure 5:**
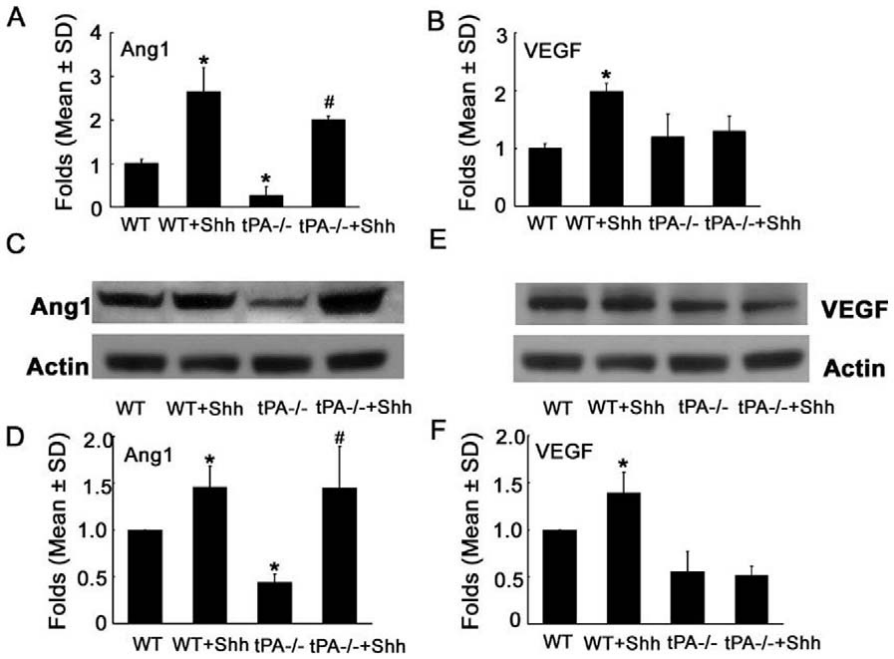
The effect of rhShh on expression of VEGF and Ang1 in wild-type and tPA−/− MBECs. Real-time RT-PCR (A, B) and Western blot (C to F) analyses showed that rhShh robustly increased Ang1 (A, C, D) and VEGF (B, E, F) expression in wild-type (WT) MBECs. Knockout of tPA (tPA−/−) substantially reduced Ang1 expression compared with wild-type MBECs (A, C, D), whereas incubation of tPA−/− MBECs with rhShh completely rescued Ang1 expression (A, C, D). However, incubation of tPA−/− MBECs with rhShh did not elevate VEGF expression, although knockout tPA significantly reduced VEGF protein levels (B, E, F). * p<0.05 versus the WT group, # p<0.05 versus the tPA−/− group. (n = 3/group). MBECs = mouse brain endothelial cells.

Previous studies have shown that Shh upregulates VEGF and Ang1 in fibroblasts and interstitial mesenchymal cells [10], [11]. We therefore examined the effect of rhShh on expression of these genes in cerebral endothelial cells. Both real-time RT-PCR analysis and Western blot showed that rhShh significantly upregulated Ang1 and VEGF expression on wild-type endothelial cells (Fig. 5A to F). Blockage of Tie2 with a neutralizing antibody against Tie2 significantly reduced rhShh-induced tube formation (3.9±1.1 vs 6.4±0.4 mm/mm^2^ in rhShh group, n = 5/group, p<0.05), while inhibition of VEGF receptor 2 (VEGFR2) with SU1498, a specific antagonist of VEGFR2, did not significantly reduce rhShh-induced tube formation (4.1±1.2 vs 6.4±0.4 mm/mm^2^ in rhShh group, n = 5, p = 0.09). Interestingly, tPA−/− endothelial cells exhibited significant reductions of Ang1 mRNA and protein (Fig. 5A, C, D), and a significant decrease in VEGF protein (Fig. 5B, E, F) compared to wild-type endothelial cells, suggesting that knockout of tPA affects Ang 1 and VEGF expression. Incubation of tPA−/− endothelial cells with rhShh robustly upregulated Ang 1, but did not alter VEGF expression (Fig. 5A to F). These data suggest that Shh-upregulated Ang1 is tPA independent, whereas upregulation of VEGF by Shh requires tPA.

## Discussion

The present study demonstrates that Shh promotes in vitro cerebral angiogenesis and that tPA and PAI-1 are involved in this process.

A prominent effect of the morphogen Shh on angiogenesis is emerging. Our observations that rhShh upregulated Gli1 and that blockage of Smo by cyclopamine abolished the effect of rhShh on the tube formation indicate that the Shh/Gli1 pathway mediates cerebral angiogenesis. These data are consistent with a previous study on mouse brain capillary endothelial cells, which shows that blockage of cyclopamine suppresses the effect of rhShh on capillary morphogenesis [13]. We noted that the dose of rhShh used in the present study (100 ng/ml) was fifty times lower than the dose in the Kanda et al study (5 µg/ml) [13]. This difference could be caused by use of different recombinant Shh, rhShh vs mouse recombinant Shh in the Kanda et al study [13]. Alternatively, primary cerebral endothelial cells used in the present study are more sensitive to exogenous Shh. A very recent study shows that rhShh at dose of 0.1 µg/ml regulates blood brain barrier (BBB) permeability in primary human brain endothelial cells [22].

Mechanisms by which Shh induces angiogenesis are incompletely understood. The present study indicates a linkage between the Shh signaling pathway and the plasminogen and plasmin system in mediating in vitro cerebral angiogenesis. The effect of exogenous Shh on tPA and PAI-1 is specific because rhShh does not alter uPA expression and blockage of the Shh pathway with cyclopamine abolishes tPA activation and restores PAI-1 expression on cerebral endothelial cells. In addition, both Shh and tPA are required to fully restore in vitro angiogenesis in tPA−/− cerebral endothelial cells. Cerebral endothelial cells are primary sources of tPA and PAI-1 [21]. tPA, a serine protease, converts plasminogen to plasmin, whereas PAI-1 neutralizes plasminogen activator activation that prevents the generation of plasmin [24]. The plasminogen and plasmin system regulates angiogenesis [16], [17]. Thus, activation of the Shh signaling pathway could regulate levels of tPA and PAI-1 on cerebral endothelial cells, which has also been recently demonstrated by us to occur in astrocytes [23], suggesting that interaction of the Shh pathway with the plasminogen/plasmin system is not cell type specific. Kanda et al previously demonstrated that Shh activates the PI3K pathway, thereby inducing angiogenesis [13]. The PI3K/Akt signaling pathway regulates tPA and PAI-1 levels [25]. Additional studies are warranted to investigate whether the PI3K/Akt pathway mediates crosstalk between the Shh pathway and the plasminogen/plasmin system.

Consistent with published data, the present study demonstrates that Shh upregulates proangiogenic factors VEGF and Ang1 on cerebral endothelial cells [13], [14]. Angiogenesis induced by tPA is mediated by elevation of VEGF [26]. Our data demonstrate that ablation of tPA substantially reduced endogenous Ang1 and VEGF expression on cerebral endothelial cells, which could account for observed impairment of tube formation. Our data further indicate that Shh regulates VEGF expression on cerebral endothelial cells in a tPA-dependent manner, as exogenous Shh could not upregulate VEGF expression on tPA−/− endothelial cells. In contrast, endogenous tPA is not required for Shh-induced Ang1 expression, since rhShh elevated Ang1 levels in tPA−/− endothelial cells, as rhShh did on wild-type endothelial cells. Studies in mesenchymal cells suggest that Shh induces Ang1 through activation of the orphan nuclear receptor, COUPTFII [27]. Thus, the present study reveals coupling of Shh and tPA in mediating cerebral angiogenesis by regulating proangiogenic factors. While our manuscript was under review, Alvarez et al showed that the Shh secreted by astrocytes interacts with cerebral endothelial cells to facilitate formation of BBB integrity and to reduce inflammation in vivo [22]. Further studies are needed to investigate whether coupling of the Shh pathway and the plasminogen/plasmin system plays a role in BBB function.

In summary, the present study demonstrates that tPA and PAI-1 are involved in Shh-induced in vitro cerebral angiogenesis, which suggests complex coupling between the Shh signaling pathway and the plasminogen/plasmin system for inducing angiogenesis.

## Materials and Methods

All experimental procedures were approved by the institutional Animal Care and Use Committee of Henry Ford Hospital. Wild-type mice (C57BL6/J, 6–8 weeks) and tPA null mice (tPA−/− mice, C57BL6 background) were purchased from the Jackson Laboratory (Bar Harbor, ME). The permit number is: #886.

### Culture of mouse brain microvascular endothelial cells

Cerebral endothelial cells were isolated from microvessels of wild-type (n = 10) or tPA knockout mice (n = 10) as previously described [28]. Briefly, mice were sacrificed and their brains were collected in RPMI 1640 medium (Invitrogen Cooperation, Carlsbad, CA) supplemented with 1% penicillin and streptomycin (Invitrogen Corporation, Carlsbad, CA). Cerebellum, white matter, meninges, and visible blood vessels of the brain were removed under a microscope. Cerebral cortex and subcortex were cut into small pieces and homogenized. Homogenates were suspended in 15% dextran (Sigma, St. Louis, MO) and centrifuged at 6,000 g for 15 min at 4°C. Pellets were resuspended and digested with 0.1% collagenase/dispase (Roche Applied Science, Penzberg, Germany) and 2% FBS (Invitrogen) in RPMI1640 medium. Digested microvessels were separated with 45% Percoll (Sigma, St. Louis, MO) at 20,000 g for 10 min at 4°C and plated into collagen I (BD Biosciences, Bedford, MA) coated plates. Cultures were maintained in endothelial growth medium. Passage 2–4 endothelial cells were employed in the present study.

### Capillary tube formation assay

Ninety-six well plates were coated with 100 µl solution containing 70% matrigel (BD Biosciences) and 30% DMEM (Invitrogen). MBECs were incubated with or without rhShh (100 ng/ml) (R&D System, Minneapolis, MN), cyclopamine (5 µM) (EMD-Calbiochem, Gibbstown, NJ), rhtPA (1 µM) (EMD-Calbiochem), or PAI-1 (10 ug/ml) (EMD-Calbiochem) for 16 h. MBECs (2×10^4^cells/well) were then seeded in the coated 96-well plates for 3–4 hours at 37°C. Formation of capillary-like networks was recorded under a 10× objective (OlympusIX71, Olympus Optical Co, LTD, Tokyo, Japan) via a color CoolSnap CCD camera (Roper Scientific Photometrics, Tucson, Arizona) and the total length of tube formation was measured by means of the microcomputer imaging device (MCID, Imaging Research, St. Catharines, Canada). Data are presented as the length in mm/mm^2^.

### Real time RT-PCR

Quantitative real-time RT-PCR was performed according to published methods [28], [29]. Briefly, total RNA from MBECs was isolated using Absolutely RNA Microprep Kit (Stratagene, La Jolla, CA) and followed by reverse transcription in accordance with the manufacturer's procedure. Real-time PCR was performed using SYBR Green PCR Master Mix (Applied Biosystems, Foster City, CA) on an ABI 7000 PCR instrument (Applied Biosystems). Three-stage program parameters provided by the manufacturer were employed, as follows: 2 min at 50°C, 10 min at 95°C, and then 40 cycles of 15 s at 95°C and 1 min at 60°C. The specificity of PCR product was verified by performing dissociation reaction plots. Each sample was tested in triplicate and data obtained from three independent experiments were used to quantify relative gene expression by the 2^−ΔΔCt^ method. Using Primer Express software (Applied Biosystems), we designed the following primers used in the present study: β-actin (forward, 5′-CCA TCA TGA AGT GTG ACG TTG-3′; reverse, 5′-CAA TGA TCT TGA TCT TCA TGG TG-3′), tPA (forward, 5′-CTG AGG TCA CAG TCC AAG CA-3′; reverse, 5′- ACA GAT GCT GTG AGG TGC AG-3′), PAI-1 (forward, 5′-GTC TTT CCG ACC AAG AGC AG-3′; reverse, 5′- ATC ACT TGG CCC ATG AAG AG-3′), uPA(forward, 5′- AGT GTG GCC AGA AGG CTC TA-3′; reverse, 5′- GCT GCT CCA CCT CAA ACT TC-3′), Ptch1(forward, 5′-GTG GAA GTT GGT GGA CGA GT-3′; reverse, 5′-TAG CGC CTT CTT CTT TTG GA-3′), Gli1(forward, 5′-TCC ACA CGC CCC CTA GTG-3′; reverse, 5′-TGG CAA CAT TTT CGG TGA TG-3′), angiopoietin 1 (Ang1, forward, 5′-CTC GTC AGA CAT TCA TCA TCC AG-3′, reverse, 5′-CAC CTT CTT TAG TGC AAA GGC T-3′), vascular endothelial growth factor (VEGF, forward, 5′-CAG GCT GCT GTA ACG ATG AA-3′; reverse, 5′-GCA TTC ACA TCT GCT GTG CT-3′).

### Western blot analysis

Western blot was performed according to published methods [30]. Briefly, equal amounts of protein (40 µg/lane) for each sample were electrophoresed through a 10% SDS-PAGE gel (Invitrogen) and subsequently electrotransferred to nitrocellulose membranes. Membranes were probed with the following primary antibodies: PAI-1(1∶500, Santa Cruz Biotechnology Inc, Santa Cruz, CA), tPA(1∶500, Santa Cruz), Ang 1 (1∶1,000, Abcam, Cambridge, MA), VEGF (1∶500, Santa Cruz), and β-actin (1∶10,000, Abcam) for 16 hrs at 4°C. For detection, horseradish peroxidase-conjugated secondary antibodies were used (1∶2000) followed by enhanced chemiluminescence development (Pierce, Rockford, IL).

### Direct casein zymography of plasminogen activator (PA) activity

Proteins from MBECs were separated by 10% SDS-PAGE and PA activity was assayed by zymography, as described previously [31], [32]. Briefly, 30 µg protein samples were mixed with the sample loading buffer without β-ME, and heating was omitted. The mixture of the lower gel (10% acrylamide) contained casein (1 mg/ml) (Sigma) and plasminogen (13 mg/ml) (American Diagnostica, Greenwich, CT) as substrates for plasmin and PA, respectively. The gel was then washed for 30 min with 2.5% Triton X-100 to remove SDS and further washed for 10 min with 0.1 M Tris buffer, pH 8. New Tris buffer was replaced and the gel was incubated for 4 hrs at 37°C to allow caseinolysis occur. On the darkly stained casein background, PA activity was visualized as light bands resulting from casein degradation. To verify loading variations, duplicate samples were used in PAI-1 Western blot. Actin levels were employed as loading control.

### Statistical analysis

Student's t-test was used to analyze data between two groups. To study the combination factor effect on angiogenesis, two-factorial design and 2-way ANOVA were considered. Statistical significance was set at p<0.05.

## Supporting Information

Figure S1 Representative real-time RT-PCR raw data of reaction (A, C) and dissociation curve (B, D) of Ptch (A, B) and Gli1 (C, D). WT = wild-type.
(TIF)
