# Supplementary figures and images for: Tissue Plasminogen Activator and Plasminogen Activator Inhibitor 1 Contribute to Sonic Hedgehog-Induced In Vitro Cerebral Angiogenesis
Source: PLoS One. 2012 Mar 14;7(3):e33444. doi: 10.1371/journal.pone.0033444 (PMC3303815; doi:10.1371/journal.pone.0033444)

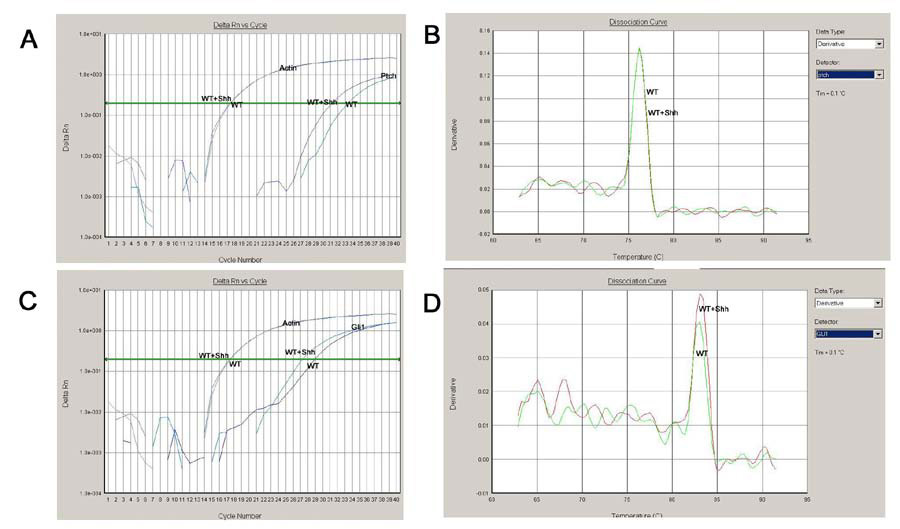

Supplement: Figure S1 — Representative real-time RT-PCR raw data of reaction (A, C) and dissociation curve (B, D) of Ptch (A, B) and Gli1 (C, D). WT = wild-type. (TIF) [file pone.0033444.s001.tif]
